# Novel Splice Variant in the *HES7* Gene in Vietnamese Patient with Spondylocostal Dysostosis 4: A Case Report and Literature Review

**DOI:** 10.3390/diagnostics15131587

**Published:** 2025-06-23

**Authors:** Ha Minh Nguyen, Nguyen Thi Kim Lien, Thinh Huy Tran, Ngoc Lan Nguyen, Suong Bang Thi Nguyen, Thi Hong Chau Bui, Nguyen Van Tung, Le Tat Thanh, Nguyen Thi Xuan, Van Khanh Tran, Nguyen Huy Hoang

**Affiliations:** 1 Biomedical Research Center, Pham Ngoc Thach University of Medicine, Ho Chi Minh 700000, Vietnam; nguyenminhha@pnt.edu.vn; 2 Institute of Biology, Vietnam Academy of Science and Technology, Hanoi 100000, Vietnam; ntkimlienibt@gmail.com (N.T.K.L.); tungnv53@gmail.com (N.V.T.); thanhlt@igr.ac.vn (L.T.T.); xuannt@igr.ac.vn (N.T.X.); 3 Center for Gene and Protein Research, Department of Molecular Pathology, Faculty of Medical Technology, Hanoi Medical University, Ministry of Health, Hanoi 100000, Vietnam; tranhuythinh@hmu.edu.vn (T.H.T.); ngoclana11108@yahoo.com (N.L.N.); 4 Biochemistry Department, University of Medicine and Pharmacy at Ho Chi Minh City, Ho Chi Minh 700000, Vietnam; suong.ntb@umc.edu.vn (S.B.T.N.); buithihongchau@ump.edu.vn (T.H.C.B.); 5 Faculty of Biotechnology, Graduate University of Science and Technology, Vietnam Academy of Science and Technology, Hanoi 100000, Vietnam

**Keywords:** *HES7* gene, novel splicing variant, spondylocostal dysostosis, Vietnamese patients, whole-exome sequencing

## Abstract

Spondylocostal dysostosis (SCDO) is a group of rare genetic disorders characterized by segmental vertebral defects and rib deformities due to congenital misalignment, fusion, or reduction in the number of ribs. The causes of the disease have been found in seven genes, including *DLL3* (SCDO1, OMIM 602768), *MESP2* (SCDO2, OMIM 608681), *LFNG* (SCDO3, OMIM 609813), *HES7* (SCDO4, OMIM 608059), *TBX6* (SCDO5, OMIM 602427), *RIPPLY2* (SCDO6, OMIM 616566), and *DLL1* (SCDO7). Among these, SCDO4, characterized by a short trunk, short neck, and mild nonprogressive scoliosis, is a rare form of reported cases. SCDO4 is identified as caused by homozygous or compound heterozygous variants in the *HES7* gene (NM_001165967.2; NP_001159439.1). This study reports a novel homozygous *HES7* splice variant (c.43-9T>A) detected in an SCDO4 patient by whole-exome sequencing and confirmed by Sanger sequencing. This variant was evaluated as an acceptor loss variant in intron 1 in the HES7 transcript by in silico analysis and was inherited from the patient’s parent. This study also reviews previous reports to provide a comprehensive overview of SCDO and help us to understand the pathogenesis to develop future treatment strategies.

## 1. Introduction

Spondylocostal dysostosis (SCDO) comprises an etiologically heterogeneous group of genetic diseases first reported by McAlister in 1973 [1]. Skeletal dysplasias occur in about 1 in 5000 births [2], while SCDO is estimated to occur in 1 in every 40,000 births and is considered a rare genetic disease [3]. SCDO is characterized by widened vertebrae, shortened trunk, and abnormal rib arrangement, resulting from disruption of the spine, ribs, associated tendons, and muscle precursor tissues due to disruption of embryonic development [4]. During this stage, pairs of vertebrae periodically bud from the anterior end of the prevertebral mesoderm (PSM) at a predetermined rate. Segment formation is a complex process controlled by the interaction of four signaling pathways, of which the NOTCH signaling pathway is key in many cellular developmental processes, including skeletal system development and bone homeostasis [5]. Notch receptors in the system are key players in regulating cell differentiation and function. Four Notch receptors that perform cell-specific functions in the skeletal system have been identified, and any alterations in these receptors result in changes in Notch signaling. Variants in the Notch signaling pathway have been reported to be associated with several bone malignancies and the development of many congenital bone disorders [6].

In recent years, clinical and molecular diagnostics advances have led to the identification of pathogenic variants in various genes associated with SCDO. Seven genes are responsible for the pathogenesis of SCDO to date, including delta-like canonical Notch ligand 3 (*DLL3*; SCDO1, OMIM 277300) [7,8], mesoderm posterior bHLH transcription factor 2 (*MESP2*; SCDO2, OMIM 608681) [9], LFNG O-fucosylpeptide 3-beta-N-acetylglucosaminyltransferase (*LFNG*; SCDO3, OMIM 609813) [10], HES family bHLH transcription factor 7 (*HES7*; SCDO4, OMIM 613686) [11], T-box transcription factor 6 (*TBX6*; SCDO5, OMIM 122600) [12], ripply transcriptional repressor 2 (*RIPPLY2*; SCDO6, OMIM 616566) [13], and delta-like canonical Notch ligand 1 (*DLL1*; SCDO7) [14]. SCOD phenotypes are divided into seven types from SCDO1 to SCDO7 based on disease-causing variants in different genes [13,15,16,17] (Table 1).

Among them, SCDO4 (OMIM 613686) is a genetically heterogeneous group of diseases characterized by abnormal spine development due to the *HES7* gene variants and was first reported by Sparrow in 2008 [11]. SCDO4 is diagnosed by clinical manifestations including intrauterine growth retardation, short stature with a short trunk, short neck, and short chest. X-ray images can detect vertebral deformities, butterfly vertebrae, and rib abnormalities. Only six variants in the *HES7* gene associated with SCDO4 have been reported, consisting of four missense variants, one frameshift variant, and one splicing variant [11,18,19,20,21] (Table 2).

This study reports a novel homozygous variant in the *HES7* gene in a SCOD4 patient that was identified by WES sequencing. In this article, we also review published studies that provide information about the etiology of this disorder and support future treatment strategies.

## 2. Case Report

**Figure 1:**
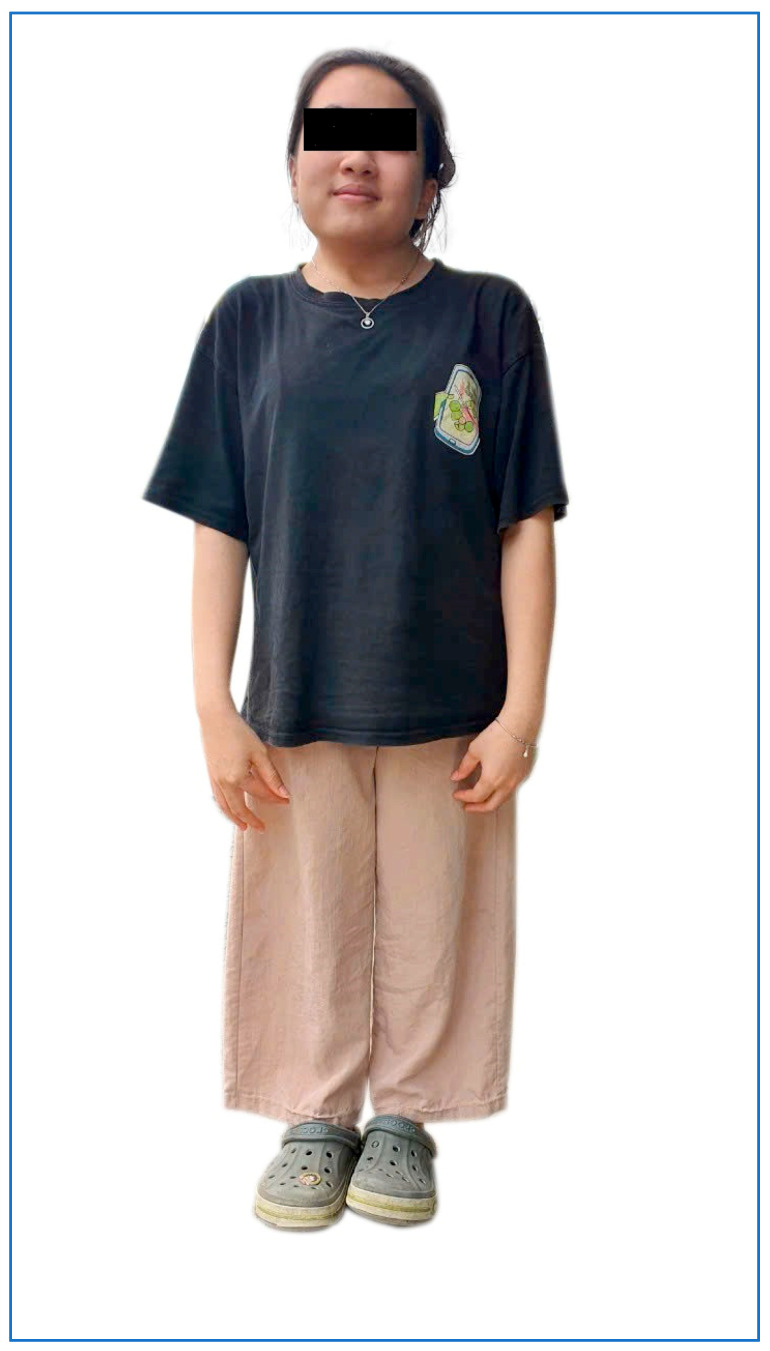
Image of patient with characteristics including short stature, short neck, and mild curved spine.

**Figure 2:**
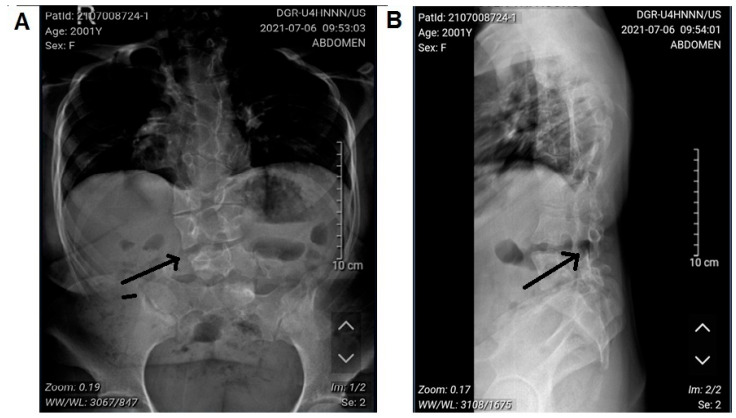
Abnormal X-ray images of the patient. (**A**). The patient’s X-ray shows a short chest with butterfly vertebrae and consolidation. (**B**). X-ray shows that the ribs are distorted and curved slightly. Black arrow points to the location of the deformed vertebrae.

A 21-year-old female patient came to Hanoi Medical University Hospital with symptoms of dwarfism, short neck, and mild scoliosis. She is 1.20 m tall and weighs 40 kg (Figure 1). X-rays showed the vertebral deformity with fused vertebrae consistent with the clinical features of SCDO4, which have been described on the OMIM database (Figure 2). However, the patient in our study did not have neural tube defects or any other abnormalities except short stature, short neck, and mild scoliosis at the hip. The patient’s nine-year-old sister has a normal phenotype (she is 1.32 m tall and weighs 30 kg), and another sibling was symptomatic (based on fetal ultrasound imaging and prenatal genotyping), so termination of pregnancy at 13 weeks was advised. Genetic analysis was carried out in the patient and members to confirm the initial diagnosis.

DNA was extracted from blood samples using a Qiagen DNA blood mini kit (QIAGEN, Hilden, Germany) and used for whole-exome sequencing (WES) on the Illumina sequencing system (Illumina, CA, USA). WES data with an average throughput depth of target regions of 150X was used for bioinformatic analysis. Software BWA (version 0.7.17, http://bio-bwa.sourceforge.net/bwa.shtml, accessed on 1 May 2025), Picard (version 2.18.2, http://broadinstitute.github.io/picard/), accessed on 1 May 2025, GATK (version 3.4, https://www.broadinstitute.org/gatk/, accessed on 1 May 2025), and SnpEff (version 4.1, http://snpeff.sourceforge.net/SnpEff.html, accessed on 1 May 2025) were used for subsequent analysis. Pathogenic variants were screened with MAF < 0.001 and evaluated based on in-silico analysis and the criteria of the American College of Medical Genetics and Genomics (ACMG) [40,41].

To validate, Sanger sequencing was carried out on an ABI PRISM 3500 (Thermo Fisher Scientific Inc., Waltham, MA, USA) and detected by BioEdit 7.2.5 software. The pathogenicity of the novel variant was predicted using in silico prediction software for the splice variant, including Ex-Skip (https://ex-skip.img.cas.cz/ , accessed on 1 May 2025), MaxEntScan (http://hollywood.mit.edu/burgelab/maxent/Xmaxentscan_scoreseq.html, accessed on 1 May 2025), Spliceailookup (https://spliceailookup.broadinstitute.org/, accessed on 1 May 2025), NetGene2 v. 2.42 (https://services.healthtech.dtu.dk/service.php?NetGene2-2.42, accessed on 1 May 2025), and Fruitfly (http://www.fruitfly.org/seq_tools/splice.html, accessed on 1 May 2025).

**Figure 3:**
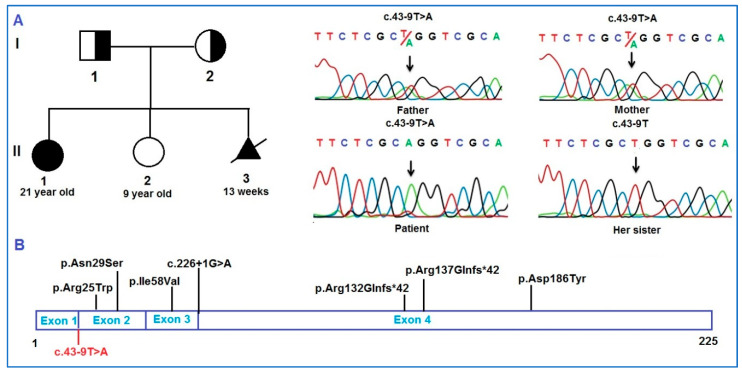
Genetic analysis of the *HES7* gene in the patient. (**A**). Pedigree of the SCDO4 patient’s family. Sanger sequencing results at the variant c.43-9T>A in the patient and the patient’s family members. (**B**). Transcripts of the *HES7* gene with the reported variants.

A novel homozygous variant (c.43-9T>A) in the *HES7* gene was identified in the patient, which was inherited from the heterozygous parents (Figure 3A,B). This novel variant is located in the splice site, is predicted to affect the splicing of HES7 mRNA (Table 3, Table S1), and therefore is considered a PS3 potent variant according to ACMG guidelines. In addition, this variant is a novel homozygous variant compared with databases such as 1000 Genomes, ExAC, and gnomAD, so it is considered a PM2-supporting and PM3-supporting variant. The patient in this report had short stature, short neck, short chest, ribs, spine, and mild scoliosis, which are the typical symptoms of SCDO4 (PP4). Based on ACMG assessment criteria, this variant has four PS3 + PM2_supporting + PM3_supporting + PP4 assessment points, so the variant is evaluated as a variant of uncertain significance (VUS) in the patient.

## 3. Discussion

In humans, congenital vertebral malformations are estimated to occur at 0.5–1 per 1000 live births [42]. Among the rare genetic disorders, the most severe is SCDO, characterized by vertebral segmental defects and congenital rib malformations due to fusion or a reduction in the number of vertebrae. Patients typically have a short trunk and scoliosis with imaging features including uneven, fused vertebrae or displaced, fused, or missing ribs [43,44]. SCDO can be inherited as an autosomal dominant or autosomal recessive, depending on the causative gene responsible for the form of the disease [15]. Although clinical and genetic knowledge regarding SCDO is improving, there remain challenges in understanding the underlying pathological mechanisms. Developing therapeutic strategies for diseases involving fetal spinal development is a promising area. However, the success of these strategies depends on one’s knowledge of the genetic causes involved in the affected embryonic developmental processes [45]. SCDO is caused by variants in genes involved in the Notch signaling pathway.

*DLL3* (delta-like canonical Notch ligand 3, OMIM 602768)—Type 1 SCDO (OMIM 277300): Type 1 SCDO is the most common form in clinical practice and is caused by variants in the *DLL3* gene [15]. The *DLL3* gene (NM_016941.4; NP_058637.1), located at 19q13.2, encodes a protein involved in somite boundary formation and cell-signaling mechanisms. Phenotypically, variants in the *DLL3* gene result in a short trunk, short neck, and moderate non-progressive scoliosis, while more significant scoliosis is rare in most affected individuals [15]. Today, *DLL3* variants, including insertion, frameshift, splice, and nonsense, leading to premature truncation or impaired protein function have been identified in association with SCDO1 in patients. Thirty-four variants in the *DLL3* gene that are associated with SCDO1 in patients have been identified (Table 2).

*MESP2* (Mesoderm posterior bHLH transcription factor 2, OMIM 605195)—Type 2 SCDO (OMIM 608681): The *MESP2* gene (NM_001039958.2; NP_001035047.1) is located on chromosome 15q26.1 and encodes the MESP2 protein, which belongs to the core-helix-loop-helix (bHLH) domain transcriptional regulatory protein family and plays an important role in rostrocaudal polarity and vertebral body formation [46,47]. The pathogenic variant of this gene is responsible for a phenotype with straight ribs and fewer fusion points than other types in patients [15]. Missense variants leading to premature stop codons causing protein truncation and other variants severely reducing protein levels have been revealed in spondylothoracic dysostosis (STD) cases, a more severe SCDO2 phenotype [9,15]. Ten pathogenic variants have been detected in patients with SCDO2 (Table 2).

*LFNG* (LFNG O-fucosylpeptide 3-beta-N-acetyl glucosaminyl transferase, OMIM 602576)—Type 3 SCDO (OMIM 609813): The *LFNG* gene, located on chromosome 7p22.3 (NM_001040167.2; NP_001035257.1), encodes single-pass type II Golgi membrane protein, a member of the glycosyltransferase superfamily that functions as a fucose-specific glycosyltransferase. Recently, *LFNG* has been reported to be associated with SCDO patients who have features of multiple severely deformed vertebrae starting from the cervical spine to the sacrum. The first individual noted had a severely shortened spine with all body vertebrae having severe segmental defects compared to other SCDO subtypes and rib malformations like SCDO types 1 and 2 [10,48]. This is followed by a report of an individual carrying a compound heterozygous variant in the *LFNG* gene with a phenotype of defects throughout the entire spine [33]. The authors evaluated the effect of the variant and confirmed that the variant significantly reduced enzyme glycosyltransferase activity [33]. To date, 17 pathogenic variants in the *LFNG* gene have been reported in patients to be associated with SCDO3 (Table 2).

*HES7* (HES family bHLH transcription factor 7, OMIM 608059)—Type 4 SCDO (OMIM 613686): The *HES7* gene (NM_032580.4; NP_115969.2), located at position 17 p13.1, encodes a protein belonging to the division enhancer transcription factor that is specifically expressed in the embryonic axial precursor mesoderm [49]. Missense variants identified resulting in significantly reduced transcriptional repressor activity in *HES7* were described in infants with short spines, segmental fusion defects, and irregularly aligned ribs, predominantly in the thoracic region [11,18]. SCDO4 is a rare SCDO type with only six pathogenic variants in the *HES7* gene reported in a small number of patients associated to date (Table 2).

*TBX6* (T-box transcription factor 6, OMIM 602427)—Type 5 SCDO (OMIM 122600): The *TBX6* gene (NM_004608.3; NP_004599.2), located on chromosome 16p11.2, encodes transcription factors that share a phylogenetically highly conserved DNA-binding domain that plays a key role in the regulation of developmental processes. Variants in the *TBX6* gene have also been identified as a cause of congenital scoliosis, a clinically distinguishable subtype of congenital scoliosis called *TBX6*-associated congenital scoliosis (TACS) [50]. SCDO-associated pathogenic variants in *TBX6* have been found as an autosomal dominant trait in a three-generation family [12,37,51]. All affected individuals were males with a similar phenotype consisting of vertebral fusion with minimal rib involvement and fused vertebral masses resulting in moderate thoracic scoliosis [19,52]. Several cases have also been reported in individuals carrying 16p11.2 deletions, nonsense variants, and frameshift variants that alter the binding site and/or affect the transcriptional regulatory activity of TBX6 [12,37,51]. Seven pathogenic variants associated with the SCDO5 type have been reported to be detected in patients (Table 2).

*RIPPLY2* (ripply transcriptional repressor 2, OMIM 609891)—Type 6 SCDO (OMIM 616566): The *RIPPLY2* gene (NM_001009994.2; NP_001009994.1) is located on chromosome 6q14.2 and encodes a protein belonging to the transcriptional repressor family that negatively regulates T-box proteins, including TBX6 [53]. The first variants were reported in two brothers with segmental defects of the cervical and thoracic vertebrae, including hemivertebral and butterfly vertebrae with obvious hunchback but normal ribs and mild thoracic scoliosis [13,15]. A variant has been shown to create a stop codon resulting in the loss of transcriptional repression activity; the other variant is a missense variant located at the mRNA splice site, but its functional consequences remain unclear [13,39,54]. However, no new variants have been reported since (Table 2).

*DLL1* (delta-like canonical Notch ligand 1, OMIM 606582) is encoded by the *DLL1* gene (NM_005618.4; NP_005609.3, 11), located on chromosome 6q27. The first report of variants in this gene was in two brothers presenting with features such as scoliosis and multiple spinal malformations with fused thoracic spines at rib positions T4T5, T6T8, and T11T12 [14]. Until now, 38 pathogenic variants in the *DLL1* gene have been reported to be associated with various diseases [55]. However, only one variant in the *DLL1* gene has been identified that causes vertebral malformations. The phenotype in patients carrying the *DLL1* variant is proposed to be a seventh form of SCD (SCDO7) [14] (Table 2).

Among them, SCDO4 is a genetically heterogeneous group of diseases presented with multiple malformations, including the fusion of various ribs, resulting in dwarfism with anomalies in the cervical region of the spine in affected individuals [17,18]. SCDO4 has many clinical manifestations in the neonatal stage, including severe chest deformity, affected lung function, and difficulty breathing with a high risk of death [56], requiring early treatment and intervention. Variants in the *HES7* gene [11], encoding a protein (bHLH) in the loop-helix superfamily of the Notch signaling pathway [17,57] and involved in the regulation of corpus cavernosum segmentation and axial skeleton formation [16,58,59], are causative of SCDO4. HES7 is expressed cyclically in the proto-astral mesoderm (PSM) over 2 h [49]. HES7 regulates the expression of the inhibitor Dusp4 in the Fgf signaling pathway and is associated with the Notch signaling pathway [11,60]. Notably, the inactivation of Notch signaling abolishes propagation but allows oscillations initiation with HES7. In contrast, the inactivation of Fgf signaling abolishes both the initiation and proliferation of HES7, thus making HES7 subject to regulation by both Fgf and Notch signaling [60]. *HES7* variants disrupted the Notch signaling pathway and vertebral segmentation, leading to phenotype in patients [61].

The variant in the *HES7* gene associated with SCDO4 was first reported in a consanguineous family of Caucasian Mediterranean origin [11]. Next, two novel variants (c.172A>G and c.556G>T) in *HES7* were identified by Sparrow et al. [18] in the patients in a family of southern European origin. In 2013, a short duplication variant (c.400_409dup10, p.Arg137Glnfs*42) was detected in a large consanguineous Arab family with seven affected individuals presenting typical features of SCDO4 [19]. In a targeted sequencing study of 73 SCDO patients, Lefebvre et al. [20] identified a novel variant p.Asn29Ser in the *HES7* gene in one patient. After that, another homozygous variant (c.226+1G>A) located in the splice region of the *HES7* gene was detected in a Chinese neonate with SCDO4 [21].

Today, six *HES7* variants are considered to be causative of SCDO4, including four missense variants, one frameshift variant, and one splice variant (Table 2). This study, a novel *HES7* variant c.43-9T>A predicted to affect *HES7* mRNA splicing (Table 3), was detected in Vietnamese patients with SCDO4. The results of the evaluation using predictive software show that there is a good match between genotype and phenotype in the patient. However, our limitation is the lack of impact assessment of variants using the mini-gene model. Evaluation using mini-gene models will help us better understand the mechanism of the role of splice variants on mRNA maturation and explain the disease mechanism in patients.

## 4. Conclusions

In summary, we have conducted the WES analysis and have identified a novel homozygous variant (c.43-9T>A) in the *HES7* gene in Vietnamese patients with SCDO4. Our results provide a comprehensive overview of SCDO and help one to understand the pathogenesis to develop future treatment strategies.

## Figures and Tables

**Table 1:** Spondylocostal dysostosis types and associated genes.

Gene	Protein	Phenotype	Number of Related Variants/ Reference
*DLL3* (AR) OMIM 602768 NM_016941.4 19q13.2	Delta protein	Spondylocostal dysostosis 1 (OMIM 277300)	34 related variants [7,8]
*MESP2* (AR) OMIM 605195 NM_001039958.2 15q26.1	Basically transcription factor	Spondylocostal dysostosis 2 (OMIM 608681)	10 related variants [9]
*LFNG* (AR) OMIM 602576 NM_001040167.2 7p22.3	Glycosyl transferase	Spondylocostal dysostosis 3 (OMIM 609813)	17 related variants [10]
*HES7* (AR) OMIM 608059 NM_001165967.2 17p13.1	Transcriptional repressor protein	Spondylocostal dysostosis 4 (OMIM 613686)	8 related variants [11]
*TBX6* (AR/AD) OMIM 602427 NM_004608.3 16p11.2	T-box transcription factor	Spondylocostal dysostosis 5 (OMIM 122600)	7 related variants [12]
*RIPPLY2* (AR) OMIM 609891 NM_001009994.2 6q14.2	Transcriptional repressor protein	Spondylocostal dysostosis 6 (OMIM 616566)	2 related variants [13]
*DLL1* (AR) OMIM 606582 NM_005618.4 6q27	Delta ligand	Spondylocostal dysostosis 7	1 related variants [14]

**Table 2:** Variants in genes associated with spondylocostal dysostosis.

Gene	Position in cDNA	Position in Protein	Reference
*DLL3*	c.535G>T	p.Glu179*	[22]
	c.621C>A	p.Cys207*	[23]
	c.661C>T	p.Arg221*	[24]
	c.712C>T	p.Arg238*	[25]
	c.805G>A	p.Gly269Arg	[26]
	c.926G>A	p.Cys309Tyr	[23]
	c.980G>A	p.Cys327Tyr	[27]
	c.1086C>A	p.Cys362*	[23]
	c.1136G>A	p.Cys379Tyr	[28]
	c.1138C>T	p.Arg380Cys	[20]
	c.1154G>A	p.Gly385Asp	[7]
	c.1164C>A	p.Cys388*	[20]
	c.1511G>A	p.Gly504Asp	[7]
	c.329delT	p.Val110Glyfs*22	[28]
	c.395delG	p.Gly132Glufs*109	[23]
	c.593insGCGGT	p.Ser198ins5	[7]
	c.599_603dupGCGGT	p.Pro202Alafs*41	[25]
	c.602_614dup13	p.Pro206Serfs*14	[23]
	c.602delG	p.Gly201Valfs*40	[23]
	c.603ins5	p.Pro206Serfs*14	[23]
	c.614ins13	p.?	[23]
	c.615delC	p.Arg205	[25]
	c.618delC	p.Cys207Alafs*34	[25]
	c.868_870+8del11	p.?	[23]
	c.945_946delAT	p.Ala317Argfs*17	[25]
	c.948_949delTG	p.Ala317Argfs*17	[23]
	c.1183_1184insCGCTGC	p.Cys395delinsSerLeuArg	[20]
	c.1238_1255dup18	p.His413_Ala418dup	[23]
	c.1256ins18	p.?	[23]
	c.1291_1307dup17	p.Pro437Thrfs*117	[25]
	c.1285–1301dup	p.?	[25]
	c.1365_1381del17	p.Cys455Trpfs*5	[23]
	c.1418delC	p.Ala473Glufs*75	[23]
	c.1440delG	p.Pro481Argfs*67	[25]
*MESP2*	c.307G>T	p.Gln103*	[29]
	c.367G>T	p.Gln123*	[20]
	c.373C>G	p.Leu125Val	[29]
	c.376G>T	p.Glu123*	[20]
	c.688C>T	p.Gln230*	[29]
	c.737G>A	p.Trp246*	[17]
	c.1166A>G	p.Glu389Gly	[17]
	c.599delA	p.Gln200Argfs*281	[17]
	c.180_193dup14	p.Glu65Alafs*60	[17]
	c.500_503dupACCG	p.Gly169Profs*199	[17]
*LFNG*	c.446C>T	p.Thr149Ile	[30]
	c.467T>G	p.Leu156Arg	[31]
	c.521G>T	p.Arg174Leu	[32]
	c.564C>A	p.Phe188Leu	[10]
	c.583T>C	p.Trp195Arg	[20]
	c.601G>A	p Asp201Asn	[33]
	c.761C>T	p.Thr254Met	[28]
	c.766G>A	p.Gly256Ser	[32]
	c.842C>G	p.Thr281Lys	[20]
	c.856C>T	p. Arg286Trp	[31]
	c.890T>G	p.Val297Gly	[34]
	c.1063G>A	p.Asp355Asn	[34]
	c.1078C>T	p.Arg360Cys	[35]
	c.44dupG	p.Ala16Argfs*135	[20]
	c.372delG	p. Lys124Asnfs*21	[33]
	c.822-5C>T		[34]
	c.863dupC	p.Asp289*	[34]
*HES7*	c.73C>T	p.Arg25Trp	[11]
	c.86A>G	p.Asn29Ser	[20]
	c.172G>A	p.Ile58Val	[18]
	c.556G>T	p.Asp186Tyr	[18]
	c.43-9T>A		This study
	c.226+1G>A		[21]
	c.400_409dup10	p.Arg137Glnfs*42	[19]
*TBX6*	c.422C>T	p.Leu141Pro	[36]
	c.449G>A	p.Arg150His	[37]
	c.661C>A	p.His221Asp	[17]
	c.699G>C	p.Trp233Cys	[36]
	c.1311G>C	p.*437Cys	[38]
	c.1148C>A	p.Ser383*	[17]
	c.994delG	p.Glu332Lysfs*166	[17]
*RIPPLY2*	c.238A>T	p.Arg80*	[39]
	c.240-4T>G		[39]
*DLL1*	c.1534G>A	p.Gly512Arg	[14]

**Table 3:** Predictions from in silico software for the splice variant c.43-9T>A.

In Silico Prediction Tools	Wildtype	Mutant	Prediction
EX-SKIP		−144.498	Exon skipping
Fruitfly	0.66	0.99	Acceptor loss
MaxEntScan	9.51	2.24	Damage variant
NetGene2	0.67	0.97	Acceptor loss
Spliceailookup	-	0.91	Acceptor loss

## Data Availability

The original contributions presented in the study are included in the article/supplementary material, further inquiries can be directed to the corresponding author.

## Supplementary Materials

The following supporting information can be downloaded at https://www.mdpi.com/article/10.3390/diagnostics15131587/s1, Table S1. Prediction using in silico tools for splice variant c.43-9T>A.
