# Supplementary material for: Novel Splice Variant in the HES7 Gene in Vietnamese Patient with Spondylocostal Dysostosis 4: A Case Report and Literature Review
Source: Diagnostics (Basel). 2025 Jun 23;15(13):1587. doi: 10.3390/diagnostics15131587 (PMC12248465; doi:10.3390/diagnostics15131587)
Supplement: Supplementary file 1 [file diagnostics-15-01587-s001.zip › diagnostics-3669589-supplementary.pdf]

Table S1. Prediction results using *in silico* tools for splicing variant c.43-9T>A

MaxEntScan

>widtype
CCTTTTCTCGC**T**GGTCGCAGATG   MAXENT: 9.51   MDD: 9.63   MM: 8.98
>mutant
CCTTTTCTCGC**A**GGTCGCAGATG   MAXENT: 2.24   MDD: 1.45   MM:.6.48

NetGene2 v. 2.4

The sequence: **wildtype** has the following composition:
Donor splice sites, direct strand

pos 5'->3'	phase	strand	confidence	5' exon intron 3'	
238	0	+	0.45	CTATTTCAGG^GTTAGTGAGG	
253	0	+	0.34	TGAGGGTACA^GTAAGCCTCC	
651	0	+	0.63	CGGCCCCAAG^GTGAGAGGCG	
1116	0	+	0.45	CGTGGAGAAG^GTGAAGGCCT	
1224	2	+	0.32	TGCGGCGCAC^GTGCGTGCGC	
**1663**	0	+	**0.97**	CCGGGACCAG^GTCAGTCCCT	H
1667	1	+	0.00	GACCAGGTCA^GTCCCTCCGC	

Acceptor splice sites, direct strand

pos 5'->3'	phase	strand	confidence	5' exon intron 3'	
1187	0	+	0.53	CTATACTCAG^GGCCGCCAGG	
1523	2	+	0.19	CCGCACTGAG^TCTCGGTCTG	
**1566**	0	+	**0.67**	CTGGTCGCAG^ATGCTCAAGC	
1575	0	+	0.17	GATGCTCAAG^CCGCTTGTGG	
1715	2	+	0.33	CGCGTCCCAG^GGCTTCCCAC	
1807	0	+	0.20	TCCATCCCAG^ACCCCCACTC	
1825	0	+	0.30	TCGCTCTCAG^AACGCGATCT	
1950	2	+	0.43	TCCCCCACAG^CCAGCCTCTG	
**2262**	0	+	**1.00**	CCGTCTGTAG^AACCTCCGGA	H
2280	0	+	0.19	GAACCCGAAG^CTGGAGAAAG	

The sequence: **mutant** has the following composition:
Donor splice sites, direct strand

pos 5'->3'	phase	strand	confidence	5' exon intron 3'	
238	0	+	0.45	CTATTTCAGG^GTTAGTGAGG	
253	0	+	0.34	TGAGGGTACA^GTAAGCCTCC	
651	0	+	0.63	CGGCCCCAAG^GTGAGAGGCG	
1116	0	+	0.45	CGTGGAGAAG^GTGAAGGCCT	
1224	2	+	0.32	TGCGGCGCAC^GTGCGTGCGC	
**1662**	0	+	**0.97**	CCGGGACCAG^GTCAGTCCCT	H
1666	1	+	0.00	GACCAGGTCA^GTCCCTCCGC	

Acceptor splice sites, direct strand

pos 5'->3'	phase	strand	confidence	5' intron exon 3'	
1187	0	+	0.53	CTATACTCAG^GGCCGCCAGG	
1523	0	+	0.20	CCGCACTGAG^TCTCGGTCTG	
**1559**	0	+	**0.97**	TTTCTCGCAG^TCGCAGATGC	
1565	0	+	0.95	GCAGTCGCAG^ATGCTCAAGC	
1574	0	+	0.17	GATGCTCAAG^CCGCTTGTGG	
1714	2	+	0.33	CGCGTCCCAG^GGCTTCCCAC	
1806	0	+	0.20	TCCATCCCAG^ACCCCCACTC	
1824	0	+	0.30	TCGCTCTCAG^AACGCGATCT	
1949	2	+	0.43	TCCCCCACAG^CCAGCCTCTG	
**2261**	0	+	**1.00**	CCGTCTGTAG^AACCTCCGGA	H
2279	0	+	0.19	GAACCCGAAG^CTGGAGAAAG	

Spliceailookup

SpliceAI scores: ?

Variant	Gene ☐ = MANE Select transcript ☐ = non-coding transcript	Δ type	Δ score ?	position ?
NM_001165967.2(HES7):c.43-9T>A ⇒ 17:8123135 A>T UCSC, gnomAD	HES7 (ENSG00000179111.9 / ENST00000541682.7 / NM_001165967.2) protein coding MANE Select transcript (minus strand) OMIM, GTEx, gnomAD, ClinGen, Ensembl, Decipher, GeneCards	Acceptor Loss	0.91	-9 bp
		Donor Loss	0.06	-298 bp
		Acceptor Gain	1.00	-2 bp
		Donor Gain	0.12	185 bp

MANE Select Transcript or All Transcripts

Pangolin scores: ?

Variant	Gene	Δ type	Δ score ?	position ?
NM_001165967.2(HES7):c.43-9T>A ⇒ 17:8123135 A>T UCSC, gnomAD	HES7 (ENSG00000179111.9 / ENST00000541682.7 / NM_001165967.2) protein coding MANE Select transcript (minus strand) OMIM, GTEx, gnomAD, ClinGen, Ensembl, Decipher, GeneCards	Splice Loss	0.96	-9 bp
		Splice Gain	0.98	-2 bp

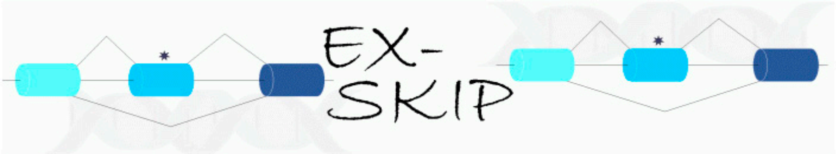

EX-SKIP - Results for submitted sequences

Seq	PESS (count)	FAS-ESS hex2 (count)	FAS-ESS hex3 (count)	IIE (count)	IIE (sum)	NI-ESS trusted (count)	NI-ESS all (sum)	PESE (count)	RESCUE-ESE (count)	EIE (count)	EIE (sum)	NI-ESE trusted (count)	NI-ESE all (sum)	ESS (total)	ESE (total)	ESS/ESE (ratio)
HES7	6	60	34	186	2781.9663	97	-144.4983	56	69	269	3487.9685	282	398.3466	383	676	0.57

Allele HES7 has a higher chance of exon skipping than allele .

Fruitfly

Donor site predictions for Human_HES7:

Start	End	Score	Exon Intron
231	245	0.96	tttcagg**gt**tagtga
246	260	0.71	gggtaca**gt**aagcct
644	658	0.99	ccccaag**gt**gagagg
1159	1173	0.65	gcgctag**gt**atctcc
1217	1231	0.66	ggcgcac**gt**gcgtgc
1656	1670	0.99	ggaccag**gt**cagtcc
1850	1864	0.79	cgggtct**gt**aagttt

Acceptor site predictions for wildtype:

Start	End	Score	Intron Exon
216	256	0.91	aggtgctaacttctatttc**ag**ggttagtgagggtacagtaa
318	358	0.79	ccaatagcctcctcctttc**ag**ctgggtcccgcctccttata
341	381	0.46	gggtcccgcctccttatac**ag**ccttacggtctgtaaaccag
400	440	0.44	cctcacttcccgcactccc**ag**gccagtttctctccttcccc
1167	1207	0.90	tatctccctttctatactc**ag**ggccgccaggtctgagcccg
1546	1586	0.66	tccttttctcgctggtcgc**ag**atgctcaagccgcttgtgga
1665	1705	0.41	cagtccctccgctagccct**ag**gtccccaagcttcccgtttc

Donor site predictions for mutant:

Start	End	Score	Exon Intron
231	245	0.96	tttcagggttagtga
246	260	0.71	gggtacagtaagcct
644	658	0.99	ccccaaggtgagagg
1159	1173	0.65	gcgctaggtatctcc
1217	1231	0.66	ggcgcacgtgcgtgc
1655	1669	0.99	ggaccaggtcagtcc
1849	1863	0.79	cgggtctgtaagttt

Acceptor site predictions for mutant:

Start	End	Score	Intron Exon
216	256	0.91	aggtgctaacttctatttcagggttagtgagggtacagtaa
318	358	0.79	ccaatagcctcctcctttcagctgggtcccgcctccttata
341	381	0.46	gggtcccgcctccttatacagccttacggtctgtaaaccag
400	440	0.44	cctcacttcccgcactcccaggccagtttctctccttcccc
1167	1207	0.90	tatctccctttctatactcagggccgccaggtctgagcccg
1539	1579	0.98	cccgctctccttttctcgcagtcgcagatgctcaagccgct
1664	1704	0.41	cagtccctccgctagccctaggtccccaagcttcccgtttc
